# The Effects of Green Tea on Diabetes and Gut Microbiome in *db*/*db* Mice: Studies with Tea Extracts vs. Tea Powder

**DOI:** 10.3390/nu13093155

**Published:** 2021-09-10

**Authors:** Guojun Wu, Anna B. Liu, Yang Xu, Ying Wang, Liping Zhao, Yukihiko Hara, Yan Y. Lam, Chung S. Yang

**Affiliations:** 1 Department of Biochemistry and Microbiology, School of Environmental and Biological Sciences and Center for Microbiome, Nutrition, and Health, New Jersey Institute for Food, Nutrition, and Health, Rutgers, The State University of New Jersey, New Brunswick, NJ 08901, USA; gary.guojun.wu@rutgers.edu (G.W.); yw652@sebs.rutgers.edu (Y.W.); liping.zhao@rutgers.edu (L.Z.); 2 Department of Chemical Biology, Ernest Mario School of Pharmacy, Rutgers, The State University of New Jersey, 164 Frelinghuysen Road, Piscataway, NJ 08854, USA; annaliu@pharmacy.rutgers.edu (A.B.L.); yangxxu@umich.edu (Y.X.); 3 Tea Solutions, Hara Office Inc., Tokyo 130-0012, Japan; ysjrhara@soleil.ocn.ne.jp; 4 Gut Microbiota and Metabolism Group, Centre for Chinese Herbal Medicine Drug Development, School of Chinese Medicine, Hong Kong Baptist University, Units 201-207, Building 15W, 15 Science Park West Avenue, Pak Shek Kok, N.T., Hong Kong, China

**Keywords:** catechins, diabetes, diet, gut microbiome, tea

## Abstract

Green tea extracts and tea catechins have been shown to prevent or alleviate diabetes. The present study tests the hypothesis that green tea leaves in powder form (GTP), which also contain fiber and other water non-extractable materials, are more effective than the corresponding green tea extracts (GTE) in impeding the development of diabetes in *db*/*db* mice. Female *db*/*db* mice were treated with a diet containing 1% of GTE, 2% of GTE, 2% of GTP (with the same catechin content as 1% GTE) or 1% GTP. The 1% GTE group had lower food intake, water consumption, body weight and fasting blood glucose levels than the control group, while 2% GTP did not have any significant effect. Dietary 1% GTE also preserved β-cell insulin secretion. However, 1% GTP increased food intake, water consumption and blood glucose levels. Microbiome analysis with 16S rRNA gene V4 sequencing showed that the gut microbiota was modified by GTE and GTP, and a few bacterial guilds were associated with blood glucose levels. In the Random Forest regression model, the leading predictor of metabolic outcome was food consumption, followed by changes in some bacterial guilds. The results illustrate the importance of food consumption and gut microbiota in affecting the progression of diabetes.

## 1. Introduction

Green tea polyphenols, mostly catechins, have been shown to mitigate or prevent metabolic diseases and obesity [1,2]. These effects have been demonstrated in rodents on a high-fat diet (HFD) or in *db*/*db* mice, a well-established model for diabetes. For example, in our previous studies, we found that supplementation with (-)-epigallocatechin-3-gallate (EGCG), the most abundant and biologically active tea polyphenol, significantly decreased body weight gain, fasting blood glucose levels and fatty liver development in mice maintained on a HFD [3,4]. In *db*/*db* mice, a standardized green tea polyphenol preparation, Polyphenon E (PPE), or EGCG decreased fasting blood glucose levels, glucose intolerance and mesenteric fat [5,6,7]. In addition to EGCG, other tea catechins include (-)-epigallocatechin (EGC), (-)-epicatechin-3-gallate (ECG), and (-)-epicatechin (EC) may also contribute to the biological activity of tea [1].

Many observational epidemiological studies and short-term randomized control trials also suggest that consumption of tea or tea polyphenols decreases the incidence of metabolic syndrome and diabetes, even though some studies did not show such an effect [8,9,10]. In most human studies, the beneficial effects in mitigating metabolic diseases were observed in individuals consuming at least 3–4 cups of green tea (600–900 mg of tea polyphenols) daily [2].

The possible mechanisms of actions of tea preparations in mitigating metabolic diseases have been studied extensively. These include a decrease in micronutrient absorption, promotion of catabolism by EGCG through metabolic regulators such as AMP-activated protein kinase (AMPK), enhancement of thermogenesis and increase in renal water reabsorption [8,11,12,13,14]. The beneficial health effects of green tea polyphenols and EGCG have also been suggested to be mediated through their modulation of gut microbiota [15]. For example, in our previous studies with *db*/*db* mice, microbiome analysis through 16S rRNA gene sequencing showed that PPE significantly altered the bacterial community structure in the cecum and colon. The changes of key bacterial phylotypes were clustered into 11 co-abundance groups or guilds, and some of these changes were correlated with the lowering of blood glucose levels [5]. However, the relative contributions of the microbiota-mediated action and other biochemical and physiological mechanisms have not been assessed. Dietary fiber and other plant materials have also been shown to have beneficial effects in preventing or alleviating diabetes through decreasing glucose absorption and promoting the growth of beneficial bacteria in the gut [16,17,18].

The aim of this study was to test the hypothesis that green tea leaves (in powder form), referred to as green tea powder (GTP), which also contains fiber and other hot water non-extractable materials in addition to catechins, would be more effective than the corresponding green tea extract (GTE) in mitigating the development of diabetes and obesity. The possible role of gut microbiota in mediating these beneficial effects was analyzed. GTP and GTE were administered through the diet to female *db*/*db* mice. Food and water consumption and body weight were carefully monitored throughout the experiment. Food consumption was found to be a major factor in affecting the progression of diabetes, which overshadows the effects of tea constituents, and microbiota changes also played an important role.

## 2. Materials and Methods

### 2.1. Tea and Diet Preparations

GTE and GTP were provided by Tea Solutions, Hara Office Inc. (Tokyo, Japan), prepared from the same batch of Japanese green tea leaves, which were manufactured by steaming fresh green tea leaves followed by rolling and drying processes. GTP was prepared by pulverizing the tea leaves to approximately 20 µm in diameter. For the preparation of GTE, the tea leaves were extracted with hot water (20 times *w*/*w* at 75 °C for 20 min) and this brewed tea solution was spray dried. Contents of catechins were determined by HPLC with procedures routinely used in our laboratory [19]. The total content of catechins in GTE was 20% (*w*/*w*, including 37.9% EGCG, 38.6% EGC, 13.5% EC and 10% ECG), and in GTP was 10.7% (including 41.6% EGCG, 34.7% EGC, 11% EC and 12.7% ECG).

Diets containing the tea preparations were prepared at Research Diets Inc. (New Brunswick, NJ, USA) by enriching the AIN-93M diet with different concentrations of GTE and GTP: 1% GTE (actually 1.07% *w*/*w*), 2% GTE (2.14% *w*/*w*), 1% GTP (*w*/*w*) and 2% GTP (*w*/*w*). Given that the catechin contents in GTE was about 2-fold of that in GTP, the 1% GTE diet and 2% GTP diets contained equal amounts of tea catechins. All diets were stored in a cold room (4 °C) until use.

### 2.2. Animal Studies

All animal experiments were performed in the animal facilities in the Department of Chemical Biology and were approved by the Institutional Animal Care and Use Committee of Rutgers University (protocol # 02-027). Five-week-old female *db*/*db* (BKS.Cg-Dock7^m^ +/+ Lepr^db^/J) and wild-type (C57BLKS/J) mice were purchased from Jackson Laboratory (Bar Harbor, ME). The mice were housed in plastic cages with corn cob bedding in a temperature (24–25 °C) and humidity (70–75%) controlled room with 12-h light-dark cycles. All mice were acclimatized for 5 days with free access to the AIN-93M diet and water. Thereafter, the *db*/*db* mice were randomized into 5 groups (10 mice per group, 5 in each cage) and received one of the following diets for 11 weeks: Control (*db*/*db* mice receiving AIN-93M diet), 1% GTE, 2% GTE, 1% GTP and 2% GTP. A group of wildtype mice were maintained on the AIN-93M diet for genotype comparison. All mice had *ad libitum* access to food and water throughout the study.

Body weight and food and water consumption were measured on Day 0 and three times a week thereafter. Fresh fecal samples were collected daily (9:00 to 9:30 a.m.) and stored at −80 °C until analysis. Fasting blood glucose levels were measured (see Section 2.3). At Days 14, 28, and 77, a fasting blood sample (100 μL) was collected from the tail vein and the serum was stored at −80 °C. The mice were euthanized at Week 11 by CO_2_ asphyxiation. Blood was collected by cardiac puncture and the serum was stored at −80 °C. Liver, pancreas and some other tissues were collected, which were either stored at −80 °C until analysis or fixed in 10% buffered formalin for at least 24 h.

### 2.3. Fasting Blood Glucose and Insulin

Fasting blood glucose levels were measured at Days 0, 7, 14, 21, 28, 49, 56, 63, 70 and 77. On the day of measurement, cage bedding was changed and the mice were fasted for 4.5 h (9:00 a.m.–1:30 p.m.) A blood sample was taken from the tail vein and blood glucose concentration was measured using the Ascensia Contour blood glucose meter (Bayer Healthcare LLC, Mishawaka, IN, USA). Serum insulin concentrations (Days 14, 28 and 77) were measured using a Rat/Mouse Insulin ELISA kit (Millipore Corporation, Billerica, MA, USA).

### 2.4. Immunohistochemistry

For immunohistochemistry (IHC), sections of pancreas (4 µm) were stained with antibodies against insulin (Cell Signaling, Danvers, MA, USA) as described previously [5]. The slides were incubated in biotin-conjugated secondary antibody (IgG, 1:200 dilution) and avidin-biotin peroxidase (Vector Laboratories, Burlingame, CA, USA) at room temperature for 1 h each. Negative controls were run in parallel without primary antibody in the incubation. The results of the immunohistochemical staining were captured by a microscope with an imaging capture system (Nikon NIS-Elements BR) and analyzed and calculated with ImageJ (Opensource Java Imaging Processing Program by NIH http://imagej.nih.gov/ij/).

### 2.5. Sample Collection and Analysis of Intestinal Microbiome

Fresh fecal samples were collected from each mouse daily at 9:00 to 9:30 a.m. into sterilized DNase/RNase-free tubes and stored at −80 °C until analysis. Genomic DNA was extracted using the QIAmp Power Fecal DNA kit (QIAGEN, Germantown, MD, USA) as per the manufacturer’s instructions with minor modifications. The 16S rRNA gene hypervariable region V4 was amplified using the modified 515F [20] and 806R [21] primers and sequenced using the Ion GeneStudio S5 (ThermoFisher Scientific, Waltham, WA, USA). Microbiome bioinformatics was performed using the QIIME 2™ platform [22]. Specifically, primers were trimmed using Cutadapt [23] and amplicon sequence variants (ASVs) were obtained by denoising using the dada2 denoise-single command with the parameters --p-trim-left 0 --p-trunc-len 215. Spurious ASVs were then removed by abundance filtering [24]. A phylogenetic tree was built using the commands alignment mafft, alignment mask, phylogeny fastree, and phylogeny midpoint-root to generate the weighted UniFrac metric. A taxonomy assignment was performed using the q2-feature-classifier plugin [25] based on the sliva database (release 132) [26]. The data were rarified to 13,000 reads/sample for subsequent analyses.

Shannon index and weighted UniFrac distance, representative metric for alpha and beta diversity respectively, were used to evaluate the overall gut microbiota structure. Non-metric multidimensional scaling (MDS) was performed using the R “vegan” package [27] to visualize differences in gut microbiota structure between treatment groups. Random forest analysis and cross-validation was performed using the R “randomForest” package [28] and the “rfcv” function respectively to test for correlations between gut microbiota composition and host phenotypes. Figures were generated using the R “ggplot2” package [29] and GraphPad Prism (version 8.0 for Windows, GraphPad Software, La Jolla, CA, USA).

ASVs shared by >40% of all samples from the *db*/*db* mice were considered prevalent and were selected for co-abundance analysis. Pairwise correlations among ASVs were calculated using the method described by Bland and Altman [30]. The correlation values were converted to a distance (1—correlation value) and the ASVs were clustered using the Ward clustering algorithm. From the top of the clustering tree, Permutational MANOVA (9999 permutations; *p* < 0.001) was used to sequentially determine whether the two clades were significantly different and cluster the prevalent ASVs into guilds [31]. Functional prediction of ASVs was performed using PICRUSt2 [32].

### 2.6. Statistical Analysis

Mouse food and water intake, body weight and fasting blood glucose levels were analyzed using one-way analysis of variance (ANOVA) followed by Tukey’s test to assess the differences among treatment groups. In addition, a 2-tailed *t*-test was used to compare a treatment group with the control group. A significant level of *p* < 0.05 was set for the test. For the gut microbiome data, multivariate association with linear models 2 (MaAsLin2) was used to examine the association between treatments and guilds/pathways after adjustment for food and water consumption. All statistical analyses were performed using R. Adjusted *p* value < 0.25 was considered significant as the default setting of MaAsLin2.

## 3. Results

### 3.1. Food and Water Consumption 

**Figure 1:**
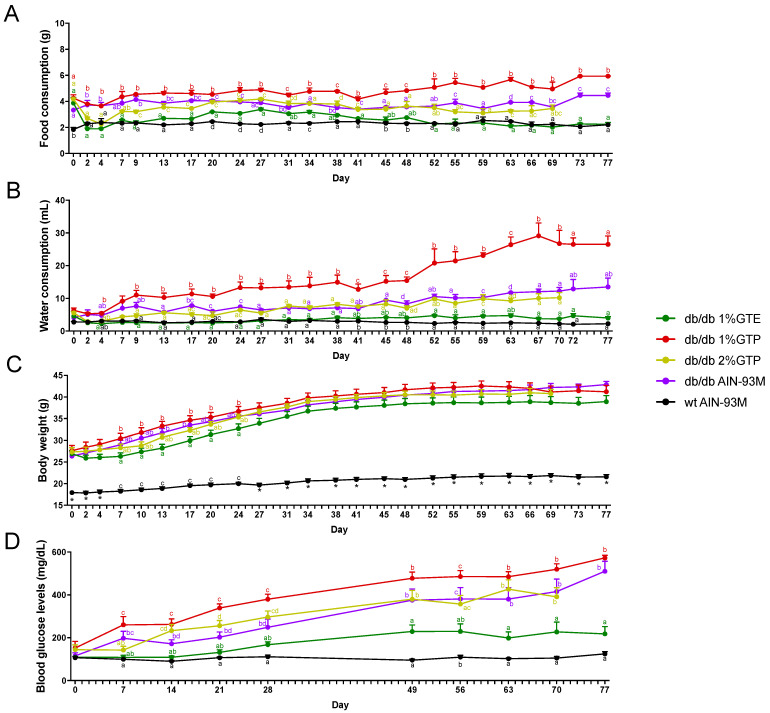
Effects of GTE (green tea extracts) and GTP (green tea leaves in powder form) treatment on mouse food consumption (**A**), water consumption (**B**), body weight (**C**) and fasting blood glucose (**D**). At each time point, ANOVA was performed followed by Tukey’s post-hoc analysis. Data points not sharing common compact letters were significantly different from each other (*p* < 0.05). The data are shown as mean ± SE. For panel (**C**), time points with asterisks show that *db*/*db* mice had a significantly higher body weight than the wildtype group and there was no difference between the 4 *db*/*db* groups. “*” showed significant difference.

Food intake, water consumption and body weight were measured three times weekly. Mice in the 2% GTE group had very low food consumption (about 30% of the amount consumed by the *db*/*db* controls) and had drastic body weight loss in the first few days of the experiment (data not shown), possibly due to bitterness and stringency of the diet. As such, they were switched to the control diet and removed from the study. For the other treatment groups, food intake in the 1% GTE and 2% GTP groups was decreased in the first 4 days (compared to Day 0), and then increased (Figure 1A). In the 1% GTE group, the daily food intake was significantly lower than the *db*/*db* control group at each time point (by ANOVA) during the period of Days 9–24. After Day 27, the 1% GTE group had a trend of decreased food intake and after Day 52, the levels of food intake were almost the same as that of the wildtype mice, which was consistently lower than the *db*/*db* control mice. On the other hand, the food intake in the 2% GTP group increased to the level of the *db*/*db* control group on Day 20 and hence were maintained at similar levels to the control group. Interestingly, food consumption in the 1% GTP group was significantly higher than that in the control group after Day 20, and after Day 45, there appeared to be an accelerated rate of food intake. Overall, the food intake in the 1% GTE, 2% GTP and 1% GTP groups were lower, the same and higher than the *db*/*db* control group, respectively.

Water consumption of the *db*/*db* control group gradually increased, while that of the wildtype mouse group remained unchanged throughout the study (Figure 1B). The effects of tea preparations on water consumption closely mirrored those observed in food intake. Mice in the 1% GTE group consumed a steadily lower level of water, with an average of 2.66 ± 0.10 mL/d (similar to that of wildtype group) for the period of Days 7–27, which was lower than the *db*/*db* control (6.88 ± 1.21 mL/d; *p* < 0.0001, *t*-test). This trend of lower level of water consumption continued to the end of the experiment, even though the values at some time points may not be significantly different from the control group by ANOVA. The water consumption of the 2% GTP group was not significantly different from the *db*/*db* control group. The 1% GTP group, however, had a higher water consumption than the *db*/*db* control group, and after Day 48, there was an accelerated increase in water intake, possibly due to polyuria caused by the development of diabetes. The daily water consumption reached an average of 27.47 ± 4.02 mL/d in Days 62–69, significantly higher than the 12.34 ± 0.92 mL/d in the *db*/*db* controls and 2.47 ± 0.18 mL/d in the wildtype group. Polyuria was evident in the 1% GTP group and the daily urine output was over 25 mL/d on Days 62–77 (data not shown).

### 3.2. Body Weight and Fasting Blood Glucose Levels

The body weights of *db*/*db* mice in all treatment groups continuously increased after adapting to the experimental diets, followed by a faster growth rate in the first 34 days and then the growth plateaued (Figure 1C). Although the ANOVA did not reveal statistical differences among the different *db*/*db* groups, the body weights of the 1% GTE group were trended lower than the *db*/*db* control groups throughout the experiment. The average body weight of mice in the 1% GTE group was significantly lower than the *db*/*db* control group on Days 10–20 (29.21 ± 3.22 g vs. 32.49 ± 3.02 g, *p* < 0.0001 by *t*-test), and this trend continued throughout the experiment; the average body weight of Days 48–77 for the 1% GTE group (38.69 ± 4.04 g) was lower than that of the control group (41.49 ± 2.98 g, *p* < 0.0001). The growth rates of mice in the 1% and 2% GTP groups were not significantly different from the *db*/*db* control group. The body weights of the wildtype mice were significantly lower and increased steadily at a much lower rate than all the *db*/*db* mice groups.

The fasting blood glucose level of the mice in the *db*/*db* control group increased almost linearly from Day 14 (171.5 ± 59.0 mg/dL) to Day 49 (375.1 ± 167.1 mg/dL) and was followed by a slower rate of increase (Figure 1D). In contrast, the blood glucose levels in the wildtype mice were in the range of 106.6 to 111.0 mg/dL. The levels in the 1% GTE group did not increase for the first 14 days, started to increase from Day 21 and reached the highest levels of 228.9 ± 96.8 mg/dL on Day 49; from that point on, the levels were significantly lower than the *db*/*db* control group. By *t*-test, the values of all data points (except Days 0 and 28) of the 1% GTE group were significantly lower than the corresponding values of the *db*/*db* control group (*p* < 0.05). The blood glucose levels in the 2% GTP group were not different from those in the *db*/*db* control group. On the other hand, the 1% GTP group showed the most rapid increase in blood glucose levels in the first 28 days, with levels significantly higher than those in the *db*/*db* control group. After Day 28, the 1% GTP group was not statistically different from the *db*/*db* control group because of the rapid increase in blood glucose level in both groups.

### 3.3. Fasting Insulin Levels and Pancreatic Insulin Immunostaining

**Figure 2:**
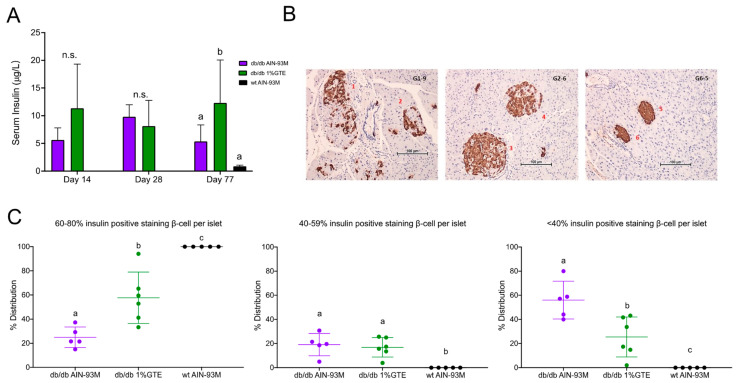
Fasting serum insulin levels and insulin immunostaining in *db*/*db* mice fed control or 1% GTE diet and the wildtype mice. (**A**) Fasting serum insulin levels on Days 14, 28, and 77. (**B**) IHC (immunohistochemistry) staining for insulin (brown color) in pancreatic β–cells in mice harvested on Day 77; from left to right, *db*/*db* AIN–93M, *db*/*db* 1% GTE, and wildtype AIN–93M. (**C**) Distribution of percent positive IHC staining cells. Data points not sharing common compact letters were significantly different from each other (*p* < 0.05).

Given the effect of 1% GTE on reducing body weight and fasting blood glucose in the *db*/*db* mice, we measured serum insulin levels to further elucidate the mechanisms by which GTE modulated glucose homeostasis. Fasting serum insulin in the mice fed with 1% GTE was significantly higher than that of the *db*/*db* controls on Day 77 (Figure 2A). Insulin immunostaining of pancreatic β-cells revealed that, compared to those in the wildtype, islets from the *db*/*db* controls on Day 77 were larger in size with uneven insulin staining, as evident by clusters of heavily stained cells as well as cells with very sparse or complete absence of a stain (Figure 2B). Hypertrophy and uneven insulin secretion associated with the *db*/*db* genotype was partly ameliorated in mice fed with 1% GTE, but most of their β-cells displayed a faint granular pattern which reflected a reduction in stored insulin granules. We further validated these histological observations by quantifying the intensity of insulin staining (Figure 2C). The number of β-cells with “60–80% positive” staining in the wildtype, *db*/*db* controls and 1% GTE group were 100%, 25%, and 58% respectively. Consequently, mice fed with 1% GTE had significantly fewer β-cells with “<40% positive” insulin staining than the *db*/*db* controls (25.5% vs. 56.0%, *p* = 0.012). These data suggest that the *db*/*db* mice over secrete insulin to compensate for the lack of insulin receptor and this wears off the β-cells as diabetes progresses. Dietary 1% GTE partly preserved the capacity of β-cells in insulin secretion.

### 3.4. Overall Gut Microbiota Structure

**Figure 3:**
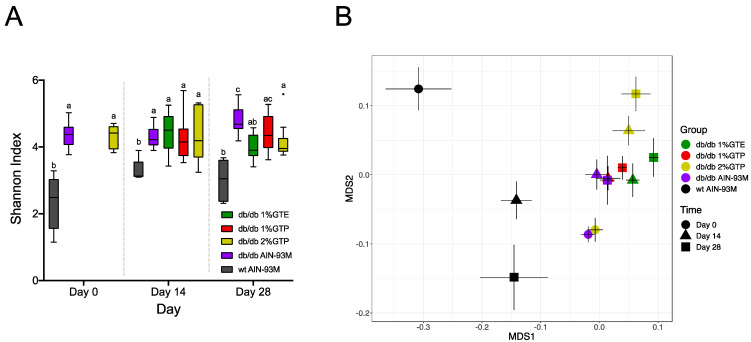
Effect of GTE and GTP treatments on the gut microbiota. (**A**) Decrease in Shannon index. Kruskal–Wallis test followed by Dunn’s post hoc was performed between groups at each time point. Data points not sharing common compact letters were significantly different from each other (*p* < 0.05). Boxes show the medians and the interquartile ranges (IQRs), the whiskers denote the lowest and highest values that were within 1.5 times the IQR from the first and third quartiles, and outliers are shown as individual points. (**B**) Alteration in gut microbiota structure. Multidimensional scaling plots for gut microbiota structure based on weighted UniFrac distance. Each data point represents the mean ordination value and the error bar represents the SEM of each group. Stress = 0.12.

To explore the role of gut microbiota in mediating the effects of tea preparations, we profiled the gut microbiota on Days 0, 14, and 28 using 16S rRNA gene V4 amplicon sequencing. A total of 867 ASVs were identified. Using Shannon index as the representative metric, gut microbiota of all *db*/*db* groups had significantly higher alpha diversity than the wildtype across all time points. Among the *db*/*db* groups, alpha diversity was similar on Day 14 but diverged on Day 28 (Figure 3A). Specifically, when compared to the *db*/*db* controls, Shannon index in the 1% GTE and 2% GTP groups was significantly lower, and a similar but non-significant trend was also observed in the 1% GTP group. Alpha diversity in all the mice treated with tea preparations, however, did not differ on Day 28.

A non-metric multidimensional scaling (MDS) plot based on weighted UniFrac distance was constructed to visualize differences in overall gut microbiota structure (Figure 3B). There was a clear separation between the wildtype and *db*/*db* mice along MDS1 and the gut microbiota of wildtype was significantly different from that of each *db*/*db* group at all time points (*p* < 0.05 by PERMANOVA test). Using the Day 0 microbiota of the 2% GTP group or *db*/*db* controls (this was adopted as Day 0 of the 1% GTP and 1% GTE groups) as baseline, all *db*/*db* groups significantly shifted along MDS2 from Day 0 to Day 14 (*p* < 0.05 by PERMANOVA test). The gut microbiota of each *db*/*db* group was also distinct from each other on Day 14 (*p* < 0.05 by PERMANOVA test), with the exception that the 1% GTP group was not different from the *db*/*db* controls (*p* = 0.0795 by PERMANOVA test). In all *db*/*db* groups, there was no further shifts in gut microbiota from Day 14 to Day 28 (*p* > 0.1 by PERMANOVA test) and all significant differences between groups remained unchanged (*p* < 0.05 by PERMANOVA test).

Next, we asked whether the tea preparations could reduce the dissimilarity of gut microbiota structure between the *db*/*db* and wildtype mice. We calculated the weighted UniFrac distance between each *db*/*db* group and the wildtype. Compared to the distance between the *db*/*db* control group and the wildtype group on Day 14, the 1% GTE and 2% GTP groups were significantly further away from the wildtype group, whereas the distance between 1% GTP and wildtype was similar to that between *db*/*db* controls and wildtype (Figure S1). Such between-group dissimilarity patterns were also observed on Day 28.

Taken together, these data suggest that the tea preparations induced rapid and distinct changes in the gut microbiota and these changes did not reduce the gut microbiota dissimilarity between the *db*/*db* and wildtype mice.

### 3.5. Guild Structure in the Gut Microbiota

Bacteria in the gut ecosystem have complex interactions and form functional groups rather than existing as isolates. Bacteria that exploit the same class of resources in a similar way can be considered as a guild, in which members typically exhibit co-abundance patterns [31]. Thus, to identify guild structures in the gut microbiota, we determined the co-abundance relationships among 106 prevalent and dominant ASVs, which were shared by >40% samples and accounted for ~90% of total bacterial abundance. These 106 ASVs were grouped into 11 different guilds.

**Figure 4:**
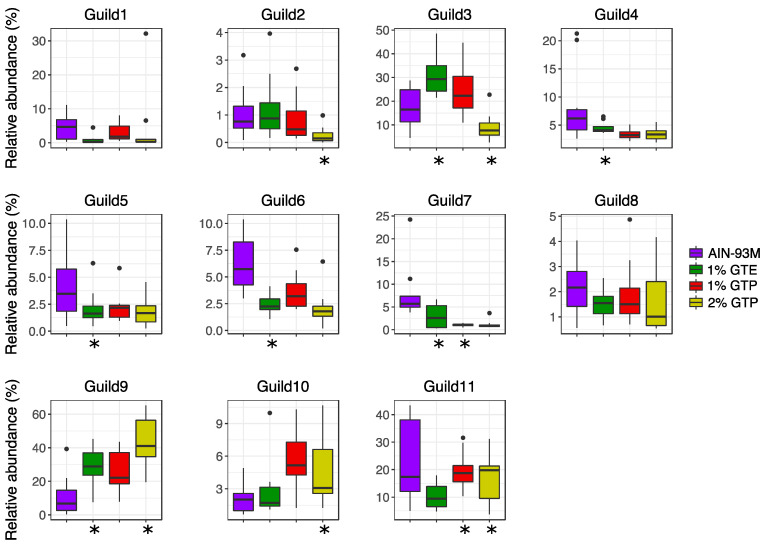
Associations between bacterial guilds and green tea preparations. The AST-transformed relative abundance of the guilds at Day 28 were used. Multivariate association with linear models 2 (MaAsLin2) was applied to explore the association between green tea compound supplements and guilds with adjustment for food and water consumption. Boxes show the medians and the interquartile ranges (IQRs), the whiskers denote the lowest and highest values that were within 1.5 times the IQR from the first and third quartiles, and outliers are shown as individual points. Compared with the *db*/*db* AIN-93M group, “*” showed significant difference (adjusted *p* < 0.25) in each treatment group.

MaAsLin2 was performed to assess the effects of tea preparations on the bacterial guilds. After adjusting for food and water consumption, the abundance of 9 of the 11 guilds was significantly different between at least one of the treatment groups and the *db*/*db* controls on Day 28 (Figure 4). Specifically, 1% GTE and 2% GTP both promoted Guild 9 (with members from *Lacotbacillus*, *Bifidobaterium*, *Parvibacter* and *Lachnospiraceae*) but differentially regulated Guild 3 (with members from *Muribaculaceae*, *Lachnospiraceae*, *Ruminiclostridium*, *Oscillibacter*, *Clostridium*, *Roseburia* and *Ruminococcaceae*; reduced by 2% GTP and increased by 1% GTE). Guilds 2, 10, and 11 were modulated by one or both of the GTP doses, whereas Guilds 4, 5, and 6 were only decreased by 1% GTE (Table S1).

**Figure 5:**
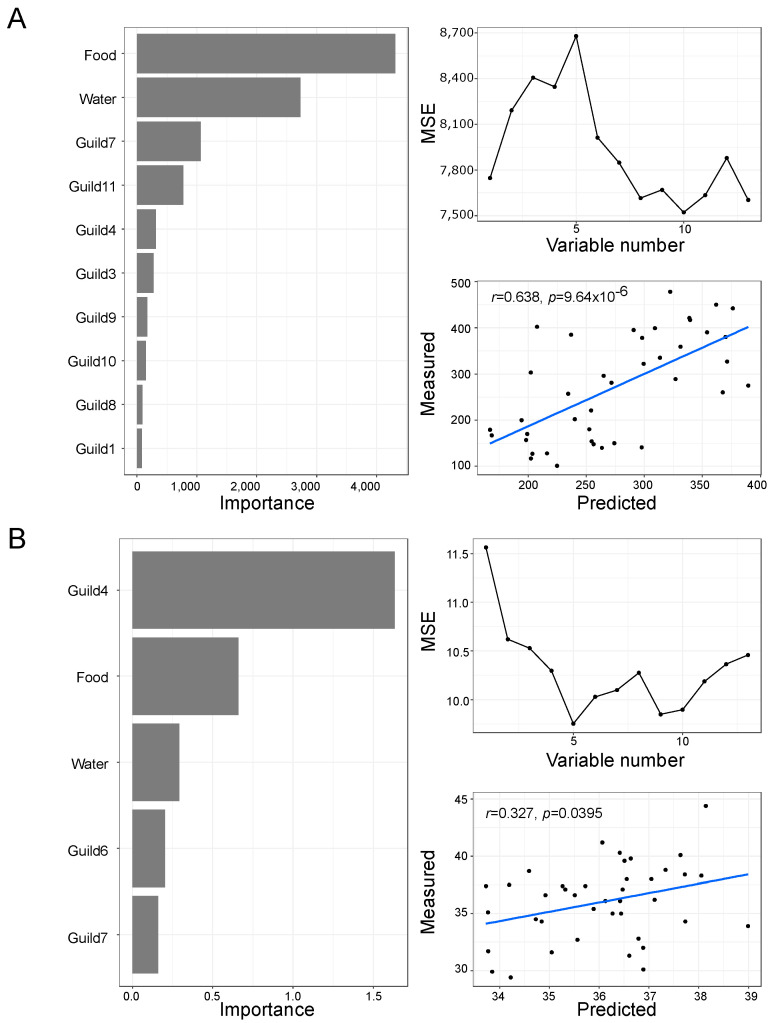
The association between gut microbiota, food and water consumption and host phenotype. (**A**) blood glucose. (**B**) body weight. Random Forest (RF) model was used to regress the blood glucose level/body weight on the guild abundance, food consumption and water consumption on Day 28. The bar charts: the RF assigns a mean error rate, or feature-importance score to each feature; this value indicates the extent to which each predictor contributes to the accuracy of the model. The curves show the number of variables and mean squared error of the corresponding model. Scatter plots of the measured values and the predicted values from leave-one-out cross-validation. Pearson correlation was applied.

Next, we explored the relevance of gut microbiota in host phenotypes by examining the associations between guilds, blood glucose and body weight using the Random Forest regression model with food and water consumption included as potential predictors. Based on the leave-one-out cross-validation and feature selection process, the best regression model, with a minimum mean square error, for blood glucose included 10 predictors (in descending order of importance in the model): food consumption, water consumption, and Guilds 7, 11, 4, 3, 9, 10, 8, and 1. The predicted blood glucose from cross-validation was significantly correlated with the measured value (r = 0.638, *p* = 9.64 × 10^−6^; Figure 5A). Among the guilds which contributed to the model, all except Guilds 1 and 8 were modulated by at least one tea preparation. The best regression model for body weight included 5 predictors, including food consumption, water consumption, and Guilds 4, 6, and 7. Based on cross-validation, there was a significant correlation between predicted and measured body weight (*r* = 0.327, *p* = 0.0395; Figure 5B). All 3 guilds included in this model were modulated by 1% GTE and Guild 7 was also modulated by 1% GTP. These data showed that the tea preparations had distinct effects on modulating the gut microbiota.

### 3.6. Functional Analysis of the Gut Microbiota

**Figure 6:**
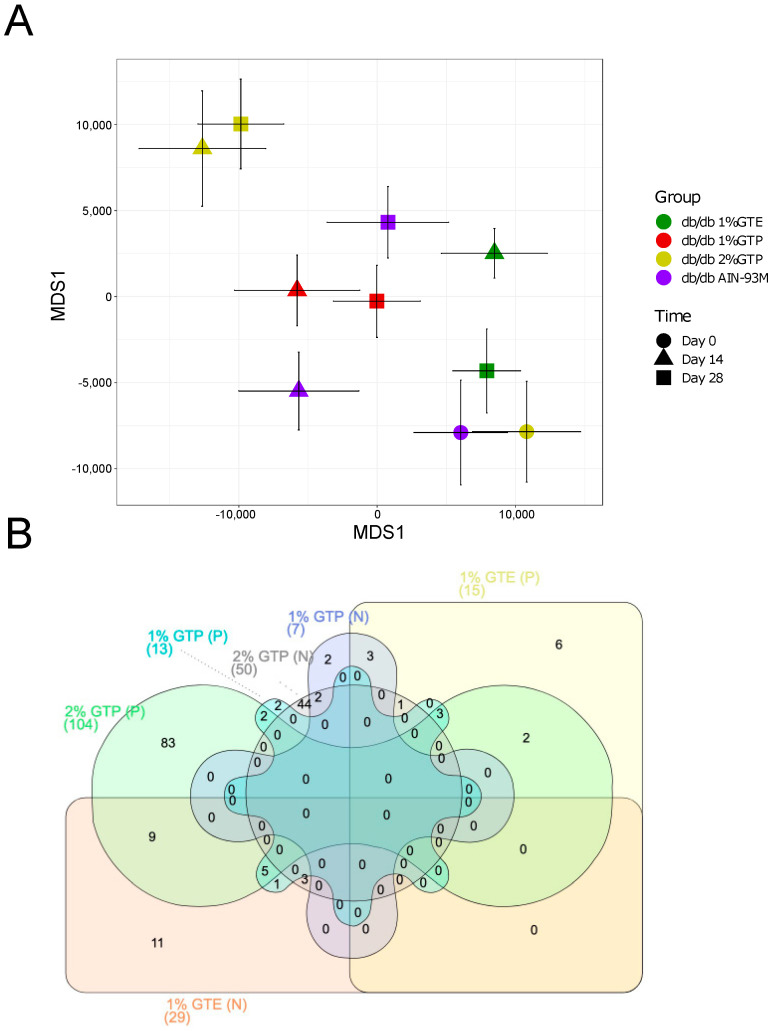
Effects of GTE and GTP treatments on the gut microbiota function. (**A**) Multidimensional scaling plots for gut microbial pathway profiles based on Euclidean distance. Each data point represents the mean ordination value and the error bar represents the SEM of each group. Stress = 0.11. (**B**) Venn diagram of the number of pathways which were associated with GTE and GTP treatment. Log-transformed abundance of the pathways from PICRUST2 at Day 28 were used. Multivariate association with linear models 2 (MaAsLin2) was applied to explore the association between green tea compound supplements and pathways with adjustment for food and water consumption. Compared with the *db*/*db* AIN-93M group, adjusted *p* < 0.25 is considered significant in each treatment group. For each treatment, (P): compared with the *db*/*db* AIN-93M group, the pathways were positively associated with the treatment; (N) compared with the *db*/*db* AIN-93M group, the pathways were negatively associated with the treatment.

Finally, we sought to identify the microbial functional signature(s) that might contribute to the effects of tea preparations on the host phenotype. Using PICRUSt2, a total of 431 microbial pathways were predicted in the gut microbiota of all *db*/*db* groups. An MDS plot based on Euclidean distance was constructed from the pathway profiles (Figure 6A). Changes in microbial functions between groups and along time paralleled that observed in gut microbiota composition. Notably, all groups had significant functional shifts from baseline to Day 14 (*p* < 0.05 by PERMANOVA test; the 1% GTP and 1% GTE groups adopted the Day 0 of *db*/*db* controls as their baseline). All groups had distinct functional profiles on Day 14, except there was no significant difference between the *db*/*db* controls and the 1% GTP group (*p* = 0.1197 by PERMANOVA test). On Day 28, there were no further changes in overall microbial function and the differences/similarities between groups remain unchanged.

We performed MaAsLin2 analysis with adjustment for food and water consumption to determine the microbial pathways modulated by the tea preparations. Compared with the *db*/*db* controls, 34, 20 and 154 pathways were significantly associated with 1% GTE (29 negative and 15 positive), 1% GTP (7 negative and 13 positive) and 2% GTP (50 negative and 104 positive) treatments, respectively (Figure 6B, Table S2). Notably, 3 pathways were more abundant in mice fed with any of the three tea preparations than the *db*/*db* controls and these were related to vanillin and vanillate degradation. Vanillic acid has been reported as one of the main catechin metabolites found in humans [33]. Unique to the 1% GTE group, the abundance of 11 pathways was lower than the *db*/*db* controls and among them, 3 pathways (super pathway of (Kdo)_2_-lipid A biosynthesis, lipid IV_A_ biosynthesis and Kdo transfer to lipid IV_A_ III (Chlamydia)) were related to lipopolysaccharide (LPS) biosynthesis. LPS has been reported as a triggering factor for obesity and diabetes [34]. These data show the microbial metabolism of tea components and the concomitant changes in microbial pathways that are known to impact host health, which further supported gut microbiota as a key mediator of the metabolic effects of green tea preparations.

## 4. Discussion

In this study, we found that dietary 1% GTE decreased body weight, fasting blood glucose levels and fasting serum insulin levels. Mice receiving 1% GTE also had higher insulin immunostaining, suggesting a protective function of GTE against the damage of pancreatic β-cells during the development of diabetes. These results indicate the beneficial effects of 1% GTE in impeding the development of diabetes. The 2% GTP and 1% GTE diets both contained approximately 0.2% catechins and these were about twice the amount in the 0.1% PPE diet used in our previous studies, which decreased fasting blood glucose and mesenteric fat [5]. EGCG is the most abundant and biologically active tea catechin [1,2]. The EGCG content of the 2% GTP diet (0.15% EGCG) is higher than that of 1% GTE diet (0.042%) or the previously used 0.1% PPE diet (0.066%). However, diet with 2% GTP supplementation did not show the beneficial effects observed with 0.1% GTE. Moreover, the 1% GTP diet increased fasting blood glucose level. Therefore, the present result cannot be explained by the activity of EGCG or total catechins. These observed results are not in line with our expectation that 2% GTP would be more effective than 1% GTE in mitigating the development of diabetes in *db*/*db* mice, because the former also contains fiber and other materials.

We found that the three tea preparation-treated groups each had distinct patterns of food intake. One of the reasons for the differences in food consumption appears to be due to the palatability of the experimental diet. This is shown by the decreased food intake in the first 4 days of the experiment in both the 2% GTP and 1% GTE groups. The mice gradually adapted to the 2% GTP and the food intake increased to the levels of the *db*/*db* control group after Day 20. The food intake of the 1% GTE group also increased after Day 4; however, it did not reach the levels of the *db*/*db* control group. The palatability of the 2% GTE diet was so low that the mice could not survive on this diet. On the other hand, the mice had little problem adjusting to the 1% GTP diet and started to show an increase in food intake after Day 4. The higher dietary intake also promoted diabetes, which in turn may contribute to the accelerated increase in food consumption after Day 55, similar to the phenomenon of diabetic polyphagia. Changes in water consumption in each group appear to follow the trends in food intake; that is, when the animals eat more, they drink more.

Microbiome analysis with 16S rRNA gene V4 sequencing showed that the gut microbiota composition and functions were significantly different among the three groups of mice treated with tea preparations. Green tea preparations modulated different bacterial guilds. After adjusting for food and water intake, we found that 1% GTP, which had the lowest concentration of catechins, only modulated two bacterial guilds, whereas more changes in bacterial guilds were found in the 1% GTE and 2% GTP groups. Notably, Guild 9, which is composed of bacterial ASVs from *Lactobacillus*, *Bifidobacterium*, *Parvibacter* and *Lachnospiraceae*, were promoted by 1% GTE and 2% GTP. *Bifidobacterium* and *Lactobacillus* have been reported to be promoted by tea polyphenol preparations from green tea, oolong tea and pu-er tea [15]. In *db*/*db* mice, supplementation with the *Lactobacillus* strains have been shown to decrease fasting blood glucose [35,36] and increases in *Bifidobacterium* strains have been associated with the attenuation of obesity and hyperglycemia [37]. Moreover, all green tea preparations decreased potentially detrimental bacterial groups, such as Guild 7, which includes *Tyzzerella* and *Turicibacter*. Bacteria in the genus *Tyzzerella* have been reported to be pro-inflammatory and related to obesity [38,39]. *Turicibacter* has been associated with colitis and obesity/diabetes in mouse models [40]. From a functional perspective, we found that the pathways related to vanillin and vanillate degradation were promoted in all the groups treated with green tea preparations. This is consistent with the known capacity of gut microbiota in degrading tea polyphenols [15], as vanillic acid is one of the main catechin metabolites [33]. Notably, among all the green tea preparations, LPS biosynthesis was only decreased in the 1% GTE group. LPS is a pro-inflammatory factor involved in the onset and progression of metabolic diseases [41]. The decrease of LPS biosynthesis suggests that the reduction of endotoxin production may mediate the beneficial effect on host phenotypes observed in the 1% GTE group.

Based on the Random Forest regression model, the most important predictor for blood glucose levels and body weight was found to be food consumption, and this was followed by the changes in different bacterial guilds such as Guilds 9 and 7. These results point to the importance of both food consumption and gut microbiota in affecting metabolic parameters. Food intake and gut microbiota interact with each other; it has been reported that fasting, caloric restriction, and hyperphagia can alter the gut microbiome [42,43,44], whereas the microbiome can affect the vagus nerve and brain regions, which play key roles in regulating feeding behaviors [45]. Thus, in addition to the palatability of the diet, the differential effect of the green tea-supplemented diets on food intake may partially result from changes in the gut microbiota, especially in the later part of the experiment. This aspect needs to be further studied. In future studies, approaches such as pair-feeding may be used to further dissect the effect of green tea and the roles of gut microbiota in modulating the host phenotypes.

## 5. Conclusions

In this study, the major determinant for the metabolic outcome is food consumption. This is especially true in the early part of the experiment. The results point to the importance of monitoring food intake in metabolic studies, and this issue was overlooked in some studies. In some experiments with animals, the differences in food intake among groups may not be large enough to be detected by ANOVA when using a small number of animals with large individual variations. It is important to carefully monitor food and water consumption throughout the experiment, as well as analyze the changes in microbiota, and take these factors into consideration when analyzing and interpreting the results.

## Data Availability

The raw gut microbiome sequencing data have been deposited to the sequence read archive at NCBI under the BioProject ID PRJNA744322.

## Supplementary Materials

The following are available online at https://www.mdpi.com/article/10.3390/nu13093155/s1, Figure S1: Increase of the Weighted UniFrac distance of *db*/*db* group to wildtype AIN-93M group, Table S1: Significantly modulated Guilds by tea preparations as compared with *db*/*db* controls, Table S2: Significantly modulated pathways by tea preparations as compared with *db*/*db* controls.

## Abbreviations

AMPK: AMP-activated protein kinase; ANOVA, analysis of variance; ASVs, amplicon sequence variants; EGCG, (-)-epigallocatechin-3-gallate; EGC, (-)-epigallocatechin, ECG, (-)-epicatechin-3-gallate; EC, (-)-epicatechin; GTE, green tea extracts; GTP, green tea powder; MaAsLin2, multivariate association with linear models 2; MDS, multidimensional scaling; PPE, Polyphenon E.
