# Supplementary material for: The Effects of Green Tea on Diabetes and Gut Microbiome in db/db Mice: Studies with Tea Extracts vs. Tea Powder
Source: Nutrients. 2021 Sep 10;13(9):3155. doi: 10.3390/nu13093155 (PMC8467950; doi:10.3390/nu13093155)
Supplement: Supplementary file 1 [file nutrients-13-03155-s001.zip › Figure S1.pdf]

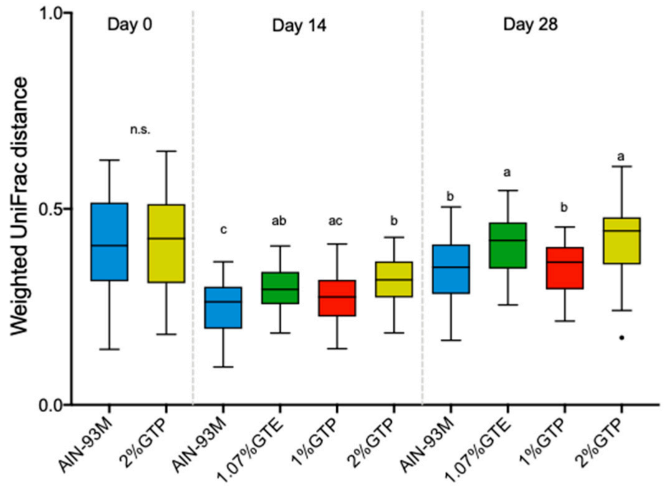

Figure S1. Increase of the Weighted UniFrac distance of *db/db* group to wildtype AIN-93M group. Kruskal-Wallis test followed by Dunn's post hoc was performed between groups at each time point. Significance ($P<0.05$) was shown as compact letter. Boxes show the medians and the interquartile ranges (IQRs), the whiskers denote the lowest and highest values that were within 1.5 times the IQR from the first and third quartiles.
